# Supplementary figures and images for: Clinical and biochemical features of atherogenic hyperlipidemias with different genetic basis: A comprehensive comparative study
Source: PLoS One. 2024 Dec 20;19(12):e0315693. doi: 10.1371/journal.pone.0315693 (PMC11661581; doi:10.1371/journal.pone.0315693)

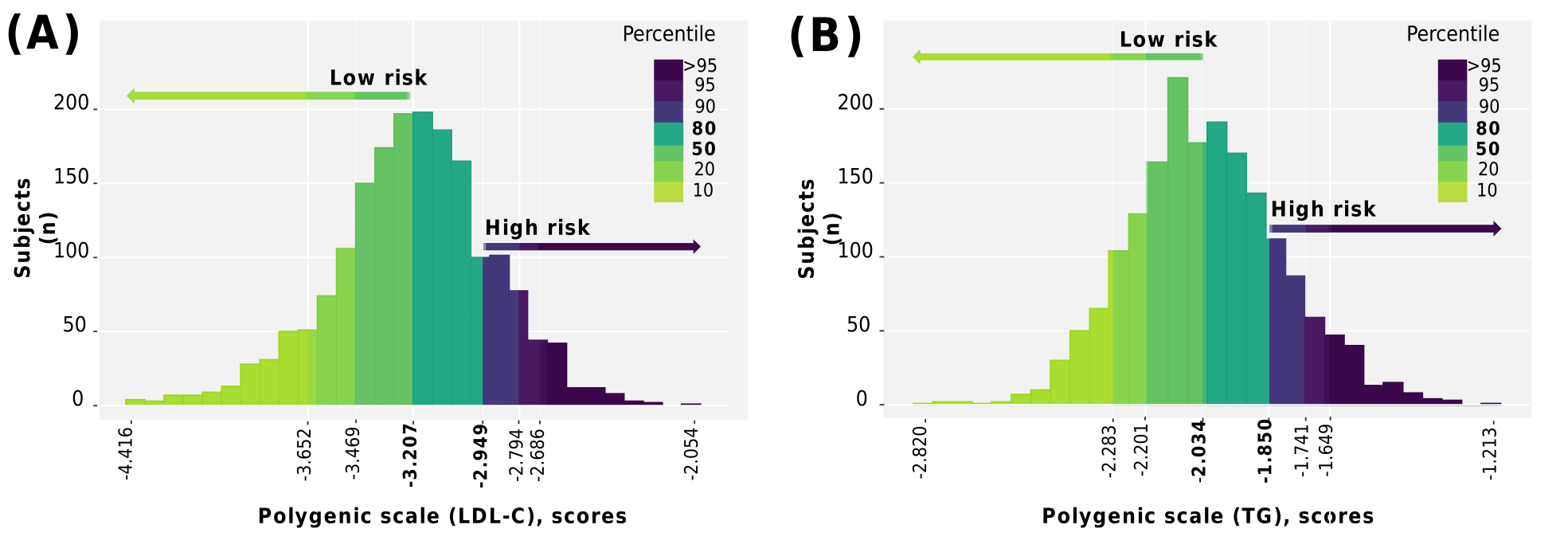

Supplement: S1 Fig — A: LDL-C levels; B: TG levels. The x-axis shows the distribution of PRS, and the y-axis shows the number of subjects. Color indicates percentiles. (TIF) [file pone.0315693.s001.tif]
