# Supplementary material for: Clinical and biochemical features of atherogenic hyperlipidemias with different genetic basis: A comprehensive comparative study
Source: PLoS One. 2024 Dec 20;19(12):e0315693. doi: 10.1371/journal.pone.0315693 (PMC11661581; doi:10.1371/journal.pone.0315693)
Supplement: S1 Table — (DOCX) [file pone.0315693.s002.docx]

**S1 Table**

| **Parameter** | **ESSE-Ivanovo sample**  **(n = 1858)** |
| --- | --- |
| Men, n (%) | 679 (36.5) |
| Age, years, Me (Q1; Q3) | 48 (37; 56) |
| BMI, kg/m^2^, Me (Q1; Q3) | 27.9 (24.4; 31.7) |
| Diabetes, n (%) | 91 (4.9) |
| Hypertension, n (%) | 1355 (72.9) |
| Coronary heart disease, n (%) | 80 (4.3) |
| Statins, n (%) | 94 (5.1)  n = 1849^a^ |
| Total cholesterol, mmol/L, Me (Q1; Q3) | 5.43 (4.69; 6.26) |
| LDL-C, mmol/L, Me (Q1; Q3) | 3.18 (2.41; 4.00) |
| HDL-C, mmol/L, Me (Q1; Q3) | 1.39 (1.19; 1.63) |
| TG, mmol/L, Me (Q1; Q3) | 1.21 (0.85; 1.79) |
| Lp(a), mg/dL, Me (Q1; Q3) | 9.5 (4.8; 27.2) |

^a^ Nine subjects had no data on lipid-lowering therapy.
