# Supplementary material for: Clinical and biochemical features of atherogenic hyperlipidemias with different genetic basis: A comprehensive comparative study
Source: PLoS One. 2024 Dec 20;19(12):e0315693. doi: 10.1371/journal.pone.0315693 (PMC11661581; doi:10.1371/journal.pone.0315693)
Supplement: S2 Table — (DOCX) [file pone.0315693.s003.docx]

| **Parameter** | **FD vs. FH** | **FD vs. Polygenic HCL** | **FD vs. Severe HCL** | **FH vs. Polygenic HCL** | **FH vs. Severe HCL** | **Polygenic vs. Severe HCL** |
| --- | --- | --- | --- | --- | --- | --- |
| Age | 0.887 | **0.041** | **0.030** | **0.044** | **0.027** | 0.372 |
| BMI | **0.015** | 0.535 | 0.813 | **< 0.001** | **0.005** | 0.728 |
| Hypertension | **0.046** | 0.413 | **< 0.001** | **< 0.001** | **< 0.001** | **0.002** |

**S2 Table**
