# Supplementary material for: Clinical and biochemical features of atherogenic hyperlipidemias with different genetic basis: A comprehensive comparative study
Source: PLoS One. 2024 Dec 20;19(12):e0315693. doi: 10.1371/journal.pone.0315693 (PMC11661581; doi:10.1371/journal.pone.0315693)
Supplement: S3 Table — (DOCX) [file pone.0315693.s004.docx]

**S3 Table**

| **Parameter** | **FD (1)** | **FH (2)** | **Polygenic**  **HCL (3)**  n = 49 | **Severe**  **HCL (1)**  n = 41 | **p - value^a^** | | | | | | **Control group** | **p - value^b^** | | | |
| --- | --- | --- | --- | --- | --- | --- | --- | --- | --- | --- | --- | --- | --- | --- | --- |
|  |  |  |  |  | 1-2 | 1-3 | 1-4 | 2-3 | 2-4 | 3-4 |  | FD | FH | Polygenic  HCL  n = 49 | Severe  HCL  n = 41 |
| **Carotid arteries** | | | | | | | | | | | | | | | |
| Maximum stenosis, %,  Me (Q1; Q3) | 32.0 (20.25; 37.75)  n = 26 | 30.0  (26.0; 40.0)  n = 61 | 28.0  (20.0; 34.0) | 28.0  (20.0; 33.0) | 1.0 | 1.0 | 1.0 | 0.540 | 0.132 | 1.0 | 0  (0; 27) | < 0.001  n = 26 | < 0.001  n = 61 | < 0.001 | < 0.001 |
| Maximum plaque height, mm,  Me (Q1; Q3) | 1.73  (1.36; 2.60)  n = 23 | 1.66  (1.31; 2.38)  n = 58 | 1.65  (1.36; 1.96) | 1.71  (1.10; 2.09) | 1.0 | 1.0 | 1.0 | 1.0 | 1.0 | 1.0 | 0  (0; 1.73) | < 0.001  n = 23 | < 0.001  n = 58 | < 0.001 | < 0.001 |
| **Femoral arteries** | | | | | | | | | | | | | | | |
| Maximum stenosis, %,  Me (Q1; Q3) | 27  (0; 36)  n = 26 | 29  (0; 36)  n = 61 | 32  (0; 68) | 21  (0;67) | 1.0 | 1.0 | 1.0 | 1.0 | 0.792 | 1.0 | 0  (0; 0) | < 0.001  n = 26 | < 0.001  n = 61 | < 0.001 | < 0.001 |
| Maximum plaque height, mm,  Me (Q1; Q3) | 2.12  (0; 2.64)  n = 23 | 2.40  (0; 3.02)  n = 59 | 1.70  (0; 2.50) | 1.29  (0; 2.39)  n=40 | 1.0 | 1.0 | 1.0 | 1.0 | 0.276 | 0.490 | 0  (0; 0) | < 0.001  n = 23 | < 0.001  n = 59 | < 0.001 | < 0.001 |

^a^p-values were obtained from linear regression with adjustments. P-values were adjusted using the Holm-Bonferroni method.

^b^p-values were obtained from Mann-Whitney test. P-values were adjusted by the Holm-Bonferroni method.
